# Disease characteristics of idiopathic transverse myelitis with serum neuronal and astroglial damage biomarkers

**DOI:** 10.1038/s41598-023-30755-0

**Published:** 2023-03-09

**Authors:** Keon-Woo Kim, Eun-Jae Lee, Sang-Yeob Kim, Hee-Jae Jung, Hyo Jae Kim, Seungmi Kim, Hyunji Kim, Dayoung Seo, Jungmin So, Jiyon Kim, Hyunjin Kim, Kwang-Kuk Kim, Young-Min Lim

**Affiliations:** 1 Department of Neurology, Asan Medical Center, University of Ulsan College of Medicine, 88 Olympic-Ro 43-Gil, Songpa-Gu, Seoul, 05505 South Korea; 2 Department of Convergence Medicine, Asan Medical Center, University of Ulsan College of Medicine, Seoul, South Korea; 3 Asan Medical Institute of Convergence Science and Technology, Asan Medical Center, Seoul, South Korea

**Keywords:** Neuroimmunology, Biomarkers

## Abstract

Despite its close association with CNS inflammatory demyelinating disorders (CIDDs), pathogenic characteristics of idiopathic transverse myelitis (ITM) remain largely unknown. Here, we investigated serum levels of neurofilament light chain (sNfL) and glial fibrillary acidic protein (sGFAP) in patients with ITM to unravel the disease characteristics of ITM. We prospectively recruited 70 patients with ITM, 62 with AQP4 + NMOSD and 85 with RRMS—including 31 patients with acute TM attacks—along with 30 HCs. We measured sNfL and sGFAP levels using single-molecular arrays and compared these levels per lesion volume between the disease groups during attacks. Compared to HCs, ITM patients showed higher sNfL and sGFAP during acute attacks (sNfL: *p* < 0.001, sGFAP: *p* = 0.024), while those in remission (sNfL: *p* = 0.944, sGFAP: *p* > 0.999) did not, regardless of lesion extents and presence of multiple attacks. ITM patients demonstrated lower sGFAP/volume (*p* = 0.011) during acute attacks and lower sGFAP (*p* < 0.001) in remission compared to AQP4 + NMOSD patients. These findings suggest that both neuronal and astroglial damages occur in patients with acute ITM attacks at a similar level to those with RRMS, distinct from AQP4 + NMOSD. However, active neuroinflammatory process was not remarkable during remission in this cohort.

## Introduction

Idiopathic transverse myelitis (ITM) refers to transverse myelitis (TM) attacks whose etiology remains unknown even after an extensive diagnostic workup^1^. Various conditions causing acute TM should be ruled out for the diagnosis of ITM, including CNS inflammatory demyelinating diseases (CIDDs), infection, malignancy, systemic autoimmune diseases, mechanical compression, spinal cord infarction and arteriovenous fistula^2,3^. Up to 40% of patients with TM attacks are diagnosed with ITM even after detailed evaluations^4^.

Notably, a number of patients with ITM could convert to other CIDDs at a later stage, such as multiple sclerosis (MS) and anti-aquaporin 4-positive neuromyelitis optica spectrum disorder (AQP4 + NMOSD)^5–7^. These close links suggest that ITM may represent an early phase of CIDDs. Meanwhile, it is also true that many ITM patients do not convert to other diseases^8–11^, suggesting that ITM may have distinct pathogenic features from other CIDDs.

Recently, serum levels of neurofilament light (sNfL), a neuronal damage marker, and glial fibrillary acidic protein (sGFAP), an astrocyte-damage marker, measured with ultrasensitive single molecular arrays have been suggested as reliable biomarkers in CIDDs^12–14^. These biomarkers could help estimate the disease activity and clinical outcome^12,15,16^ and unravel the pathogenetic characteristics of neurological disorders^17,18^. Investigating these markers would elucidate critical pathogenetic features of ITM in terms of persistence of inflammation after clinical attacks, major types of damaged cells, and degree of damage, as compared to other CIDDs.

In the present study, we aimed to examine the pathogenetic characteristics in patients with ITM by investigating sNfL and sGFAP. In this regard, we first evaluated whether sNfL and sGFAP reflect disease activity and severity in patients with ITM. Subsequently, we explored the pathogenetic characteristics of ITM, comparing sNfL and sGFAP levels between patients with ITM and those with other CIDDs—relapsing–remitting MS (RRMS) and AQP4 + NMOSD—during acute attack and remission phases.

## Results

### Baseline demographics and disease characteristics

**Figure 1:**
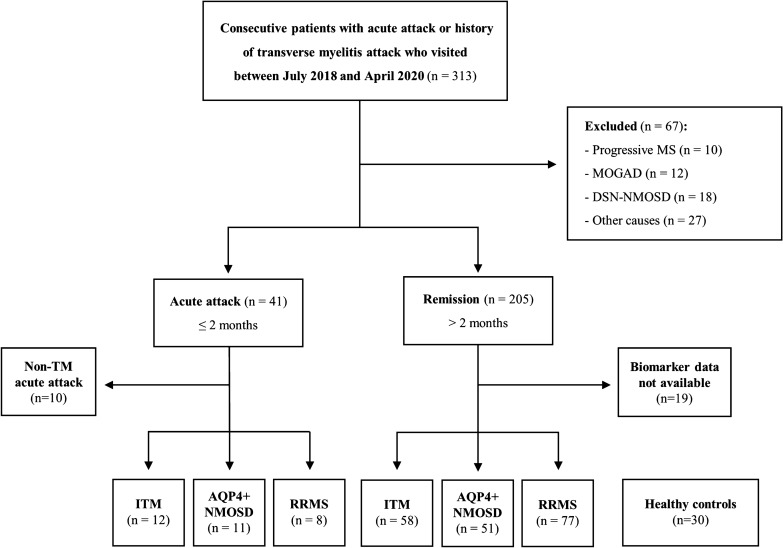
Flowchart of patient distribution. The chart shows the distribution of 313 consecutive patients who visited our center during the study period. “Acute attack” stands for a clinical attack within the last two months. ITM: idiopathic transverse myelitis; AQP4 + NMOSD: aquaporin-4 antibody-positive neuromyelitis optica spectrum disorder; MS: multiple sclerosis; RRMS: relapsing–remitting multiple sclerosis; MOGAD: myelin oligodendrocyte glycoprotein antibody-associated disorder; DSN-NMOSD: double seronegative neuromyelitis optica spectrum disorder.

During the study period, a total of 313 patients with acute attacks or a history of TM attacks visited our center, and we enrolled 70 patients with ITM, 62 with AQP4 + NMOSD, 85 with RRMS and 30 HCs (Fig. 1). Table 1 shows the demographic and clinical data of the patients and HCs. Patients with ITM were more likely to be male than those with other diseases and HCs. ITM patients showed lower Expanded Disability Status Scale (EDSS) score, shorter length of segments involved and lower prevalence of CSF pleocytosis than AQP4 + NMOSD patients. When compared to RRMS patients, they showed older age, longer length of segments involved and lower prevalence of increased IgG index. None of the patients with ITM enrolled in this study were re-diagnosed with other CIDDs until December 2021 (median follow-up duration: 38 months).

**Table 1:** Demographic and clinical features of patients and healthy controls.

	ITM (n = 70)	AQP4 + NMOSD (n = 62)	RRMS (n = 85)	HC (n = 30)	*p* value			
					Multiple comparison	ITM vs. NMOSD	ITM vs. RRMS	ITM vs. HCs
Age, year	56 [45–63]	55 [48–61]	47 [36–57]	51.0 [32.0–65.0]	**0.001**	> 0.999	**0.002**	> 0.999
Age at onset, year	49 [41–58]	46 [37–51]	33 [26–44]	–	** < 0.001**	0.120	** < 0.001**	–
Male	48 (68.6%)	4 (6.5%)	20 (23.5%)	12 (40.0%)	–	** < 0.001**	** < 0.001**	**0.014**
Acute attack phase (< 2 months)	12 (17.1%)	11 (17.7%)	8 (9.4%)	–	–	> 0.999	0.228	–
Interval since the last attack, months	36.4 [5.0–90.8]	24.7 [2.9–50.3]	28.1 [12.3–92.4]	–	0.072	–	–	–
EDSS score	2.5 [1.0–3.5]	3.5 [2.0–4.5]	2.0 [1.0–4.0]	–	**0.003**	**0.041**	> 0.999	–
Multiple clinical attacks	11 (15.7%)	50 (80.6%)	73 (85.9%)		–	** < 0.001**	** < 0.001**	–
Annual relapse rate, /year	–	0.50 [0.30–1.00]	0.33 [0.18–0.67]	–	–	–	–	–
Number of segments involved (last attack)	2.0 [1.0–4.0]	4.0 [3.0–5.5]	1.5 [1.0–2.0]	–	** < 0.001**	** < 0.001**	**0.040**	–
Autoantibodies	0 (0.0%)	30 (48.4%)	9 (10.6%)	–	–	** < 0.001**	**0.004**	–
Anti-nuclear (ANA)	0 (0.0%)	24 (38.7%)	7 (8.2%)	–	–	** < 0.001**	**0.017**	–
Anti-SS-A/SS-B	0 (0.0%)	24 (38.7%)	4 (4.7%)	–	–	** < 0.001**	0.127	–
Anti-neutrophil cytoplasmic (ANCA)	0 (0.0%)	4 (6.5%)	1 (1.2%)	–	–	**0.046**	> 0.999	–
CSF findings								
Pleocytosis	10/36 (27.8%)	32/51 (62.7%)	16/70 (22.9%)	–	–	**0.002**	0.637	**–**
Increased protein	13/36 (36.1%)	29/51 (56.9%)	14/70 (20.0%)	–	–	0.081	0.099	**–**
Positive oligoclonal band	0/22 (0.0%)	3/27 (11.1%)	4/42 (9.5%)	–	–	0.242	0.290	–
Increased IgG index (> 0.65)	4/31 (12.9%)	1/41 (24.4%)	28/64 (43.8%)	–	–	0.158	**0.003**	–
Treatment at sampling	8 (11.4%)	58 (93.5%)	76 (89.4%)	–	–	** < 0.001**	** < 0.001**	–
Corticosteroid	8 (11.4%)	30 (48.4%)	11 (12.9%)	–	–	–	–	–
Azathioprine	–	27 (43.5%)	–	–	–	–	–	–
Mycophenolate mofetil	–	3 (3.5%)	–	–	–	–	–	–
Rituximab	–	8 (9.4%)	–	–	–	–	–	–
Interferon beta	–	–	24 (28.2%)	–	–	–	–	–
Glatiramer acetate	–	–	3 (3.5%)	–	–	–	–	–
Dimethyl fumarate	–	–	8 (9.4%)	–	–	–	–	–
Teriflunomide	–	–	22 (25.9%)	–	–	–	–	–
Fingolimod	–	–	2 (2.4%)	–	–	–	–	–
Alemtuzumab	–	–	12 (14.1%)	–	–	–	–	–

Values represent medians with interquartile ranges or counts with percentages. *p* values for pairwise comparisons are demonstrated only for ITM-including pairs. *p* values with statistical significance (*p* < 0.05) are presented in bold.

ITM: idiopathic transverse myelitis; AQP4 + NMOSD: aquaporin-4 antibody-positive neuromyelitis optica spectrum disorder; RRMS: relapsing–remitting multiple sclerosis; HC: healthy control; EDSS: Expanded Disability Status Scale.

### Serum biomarker levels in ITM patients and healthy controls

**Figure 2:**
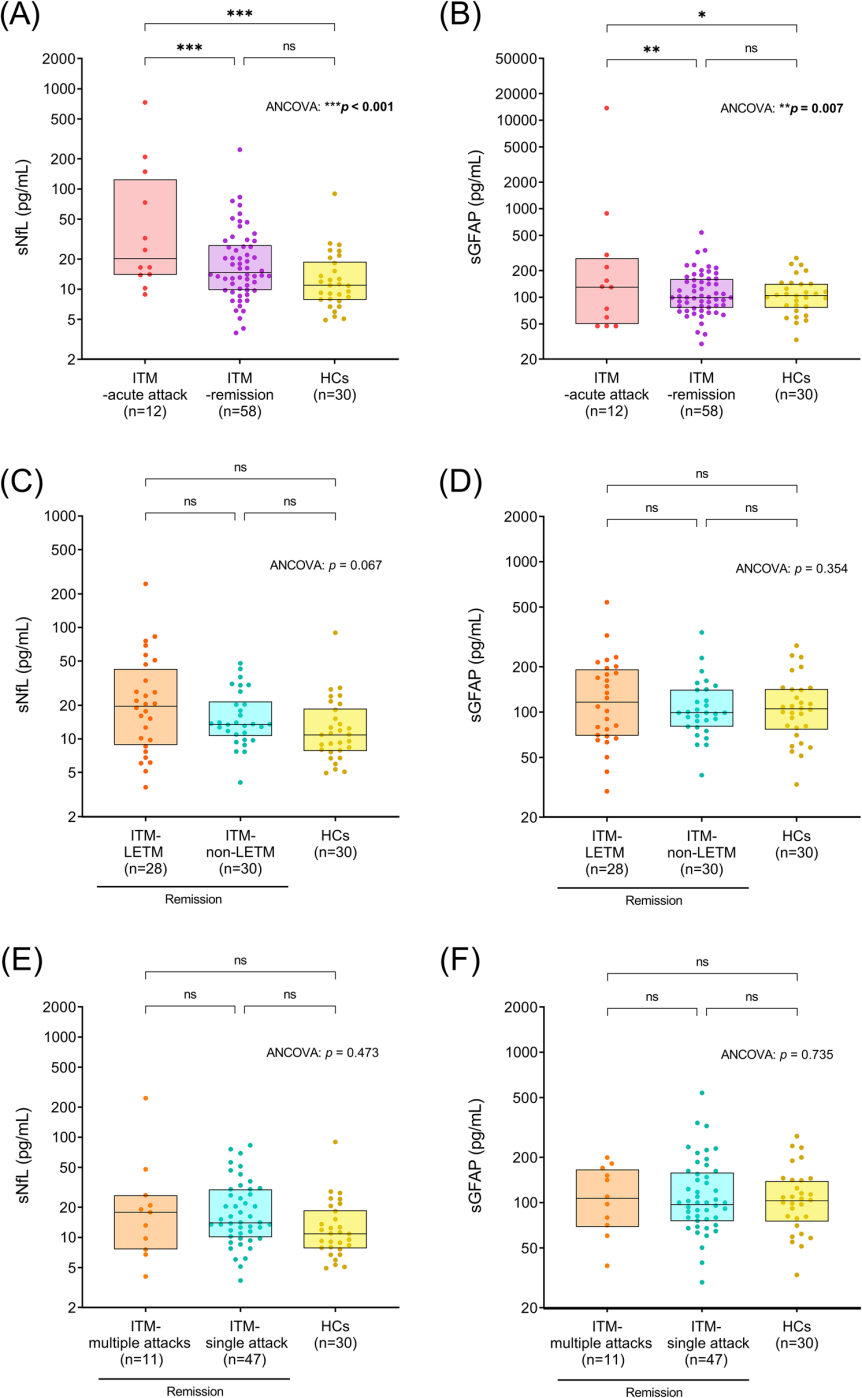
Serum biomarker levels in ITM patients and healthy controls. (**A**,**B**) sNfL and sGFAP levels of ITM patients (n = 70) and healthy controls (n = 30) were compared. “Acute attack” (n = 12) stands for a clinical attack within the last two months. (**C**,**D**) sNfL and sGFAP levels were compared between ITM patients in the remission phase who experienced LETM (n = 28) and those who did not experience LETM (n = 30). (**E**,**F**) sNfL and sGFAP levels were compared between ITM patients in the remission phase who experienced a single attack (n = 47) and those who experienced multiple attacks (n = 11). The boxes represent medians and interquartile ranges (IQR). Age was considered as a covariate in the analyses. *p* values with statistical significance (*p < 0.05, **p < 0.01 and ***p < 0.001) are presented in bold. sNfL: serum neurofilament; sGFAP: serum glial fibrillary acidic protein; ITM: idiopathic transverse myelitis; HCs: healthy controls; LETM: longitudinally extended transverse myelitis; ANCOVA: analysis of covariance.

sNfL levels were significantly higher in ITM patients in the acute attack phase (median [IQR]: 20.57 [13.94–129.95] pg/mL) than in those in the remission phase (14.64 [9.81–27.52] pg/mL, *p* < 0.001) and HCs (10.92 [7.83–18.81] pg/mL, *p* < 0.001). Of note, ITM patients in the remission phase and HCs showed comparable sNfL levels (Fig. 2A, p = 0.944). sGFAP levels were also significantly higher during the acute attack phase of ITM (130.76 [50.47–280.17] pg/mL) compared to the remission phase (99.71 [76.48–161.54] pg/mL, *p* = 0.005) and HCs (104.94 [76.48–142.27] pg/mL, *p* = 0.024), while ITM patients in the remission phase and HCs again showed similar levels (Fig. 2B, p > 0.999). When ITM patients in remission were divided into an LETM and a non-LETM group and then compared with HCs, none showed a significant difference in sNfL and sGFAP levels (sNfL: Fig. 2C, p = 0.067, sGFAP: Fig. 2D, p = 0.354). In addition, ITM patients in remission who experienced multiple attacks and those who did not both showed comparable sNfL and sGFAP levels compared to HCs (sNfL: Fig. 2E, p = 0.473, sGFAP: Fig. 2F, p = 0.735).

### Serum biomarker levels in ITM patients and their associations with clinical parameters

**Figure 3:**
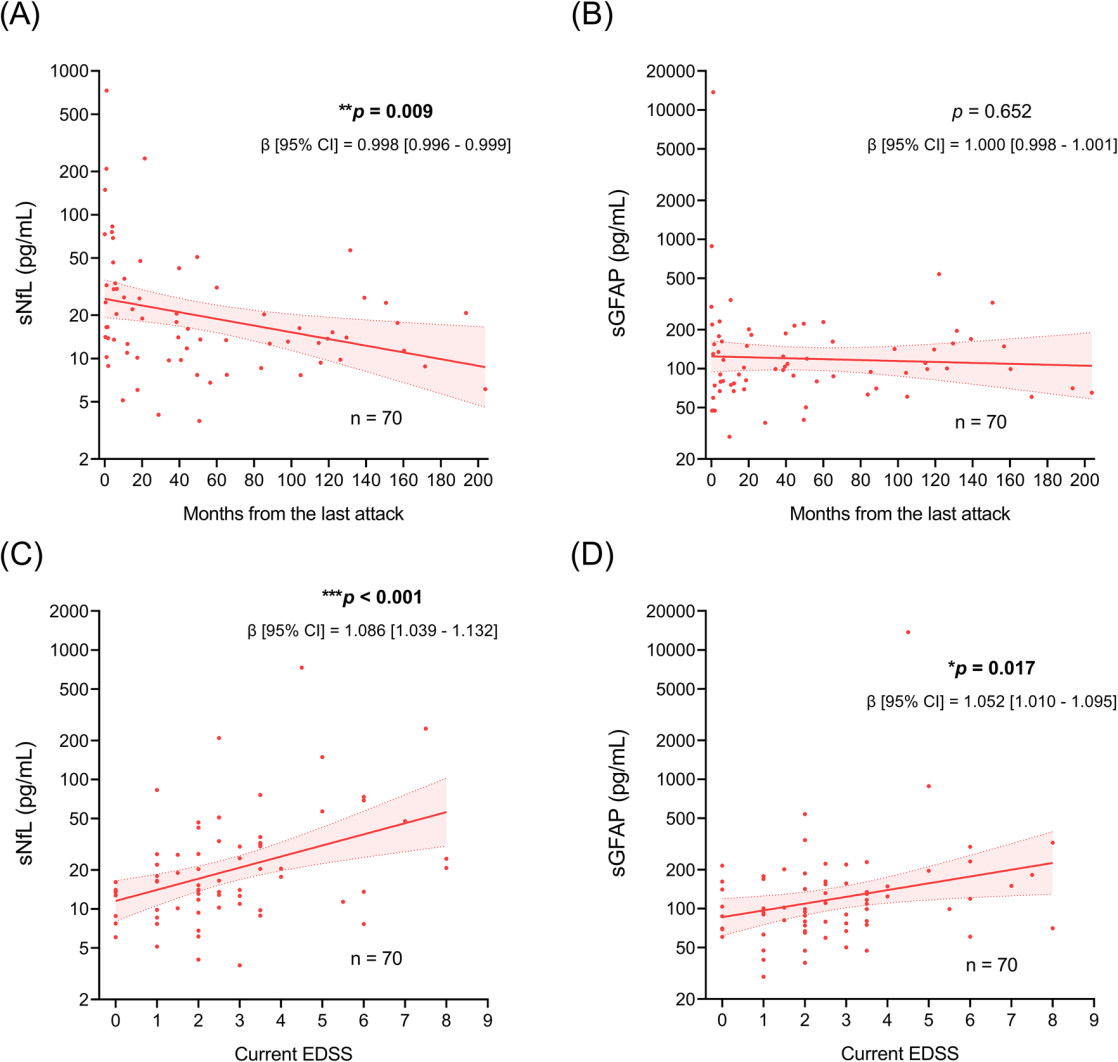
Associations between serum biomarkers and clinical parameters. (**A**,**B**) Associations between serum biomarker levels and attack-to-blood sampling intervals in ITM patients (n = 70). (**C**,**D**) Associations of serum biomarkers with EDSS scores in ITM patients. Regression lines and *p* values are derived from simple linear regression models. Translucent bands indicate 95% CI. *β* reflects multiplicative effects because the endpoint sNfL and sGFAP were log-transformed. *p* values with statistical significance (*p < 0.05, **p < 0.01 and ***p < 0.001) are presented in bold. sNfL: serum neurofilament; sGFAP: serum glial fibrillary acidic protein; ITM: idiopathic transverse myelitis; EDSS: Expanded Disability Status Scale; CI: confidence interval.

We further explored which clinical variable independently determines serum biomarker levels in patients with ITM. sNfL showed a significant decrement over time from the advent of the clinical attack (Fig. 3A, p = 0.009), while sGFAP did not show such a significant change (Fig. 3B, p = 0.652). Both sNfL (Fig. 3C, p < 0.001) and sGFAP (Fig. 3D, p = 0.017) demonstrated significant positive correlations with higher EDSS scores. In the multivariable analyses (Table 2), sNfL still showed a significant association with higher EDSS scores (*p* = 0.007), while the association between sGFAP levels and EDSS score was not significant (*p* = 0.133). Acute attack phase (sNfL: *p* = 0.003, sGFAP: *p* = 0.023), older patient age (sNfL: *p* = 0.003, sGFAP: *p* = 0.004) and more severe extent of myelitis (sNfL: *p* = 0.041, sGFAP: *p* = 0.006) were independent factors associated with sNfL and sGFAP in the multivariable models. Taken together, patient age, disease activity and myelitis extent were common independent contributors of sNfL and sGFAP in ITM patients. sNfL was also independently associated with disability, while sGFAP was not.

**Table 2:** Univariable and multivariable models for serum biomarkers in ITM patients.

Variable (no. of samples)	Serum NfL					Serum GFAP				
	sNfL, pg/mL [IQR]	Univariable		Multivariable		sGFAP, pg/mL [IQR]	Univariable		Multivariable	
		*β* [95% CI]	*p* value	*β* [95% CI]	*p* value		*β* [95% CI]	*p* value	*β* [95% CI]	*p* value
Age (70)	–	1.009 [1.002–1.017]	**0.019**	1.011 [1.004–1.019]	**0.003**	–	1.008 [1.002–1.015]	**0.017**	1.010 [1.003–1.017]	**0.004**
Sex										
Male (48)	14.04 [9.47–26.48]	0.740 [0.533–0.947]	**0.014**	0.868 [0.683–1.053]	0.160	93.49 [67.52–149.40]	0.758 [0.579–0.938]	**0.009**	0.866 [0.696–1.036]	0.120
Female (22)	17.25 [13.50–43.99]	–	–	–	–	133.03 [95.71–190.30]	–	–	–	–
Disease activity										
Acute attacks (12)	20.57 [13.94–129.95]	1.329 [1.076–1.583]	**0.012**	1.387 [1.133–1.641]	**0.003**	130.76 [50.47–280.17]	1.209 [0.982–1.437]	0.070	1.271 [1.038–1.504]	**0.023**
Remission (58)	14.64 [9.81–27.52]	–	–	–	–	99.71 [76.48–161.54]	–	–	–	–
EDSS score (70)	–	1.086 [1.039–1.132]	** < 0.001**	1.062 [1.018–1.107]	**0.007**	–	1.052 [1.010–1.095]	**0.017**	1.031 [0.990–1.072]	0.133
Number of segments involved (70)	–	1.037 [1.001–1.073]	**0.043**	1.032 [1.001–1.062]	**0.041**	–	1.043 [1.012–1.074]	**0.007**	1.039 [1.011–1.067]	**0.006**
Multiple ITM attacks										
Yes (8)	17.95 [7.66–26.48]	0.950 [0.675–1.225]	0.718	0.943 [0.710–1.177]	0.629	108.92 [70.44–169.54]	0.938 [0.697–1.178]	0.607	0.951 [0.737–1.166]	0.651
No (48)	16.12 [10.96–31.10]	–	–	–	–	100.33 [74.08–162.27]	–	–	–	–

Linear regression models were used to investigate the associations between serum biomarker levels and clinical variables. “Acute attack” stands for a clinical attack within the last two months. Values represent regression coefficient *β* with 95% CI. *β* reflects multiplicative effects because the endpoint sNfL and sGFAP were log-transformed. *p* values with statistical significance (*p* < 0.05) are presented in bold.

ITM: idiopathic transverse myelitis; sNfL: serum neurofilament; sGFAP: serum glial fibrillary acidic protein; IQR: interquartile range; EDSS: Expanded Disability Status Scale; LETM: longitudinally extended transverse myelitis; CI: confidence interval.

### Comparison of serum biomarker levels between different CNS inflammatory demyelinating diseases in the acute attack phase

Next, we investigated whether the pattern of changes in serum biomarkers during acute attack phases differs from that observed in the other CIDDs. Demographic and clinical data of the patients with acute TM attacks (12 with ITM, 11 with AQP4 + NMOSD and eight with RRMS) are presented in Table 3. The patients with ITM had a longer interval between the sampling and the last attack and lower treatment-receiving rate than those with AQP4 + NMOSD. They did not show significant differences in variables compared to patients with RRMS. We further explored whether TM lesion volumes correlate with serum biomarker levels and found that both sNfL and sGFAP levels tended to increase with higher TM lesion volumes, especially for the sGFAP in the ITM and AQP4 + NMOSD groups (Fig. S1). Given that myelitis lesion extent may affect serum biomarker levels, we additionally compared lesion volume-divided indices of serum biomarkers (sNfL/volume and sGFAP/volume).

**Figure 4:**
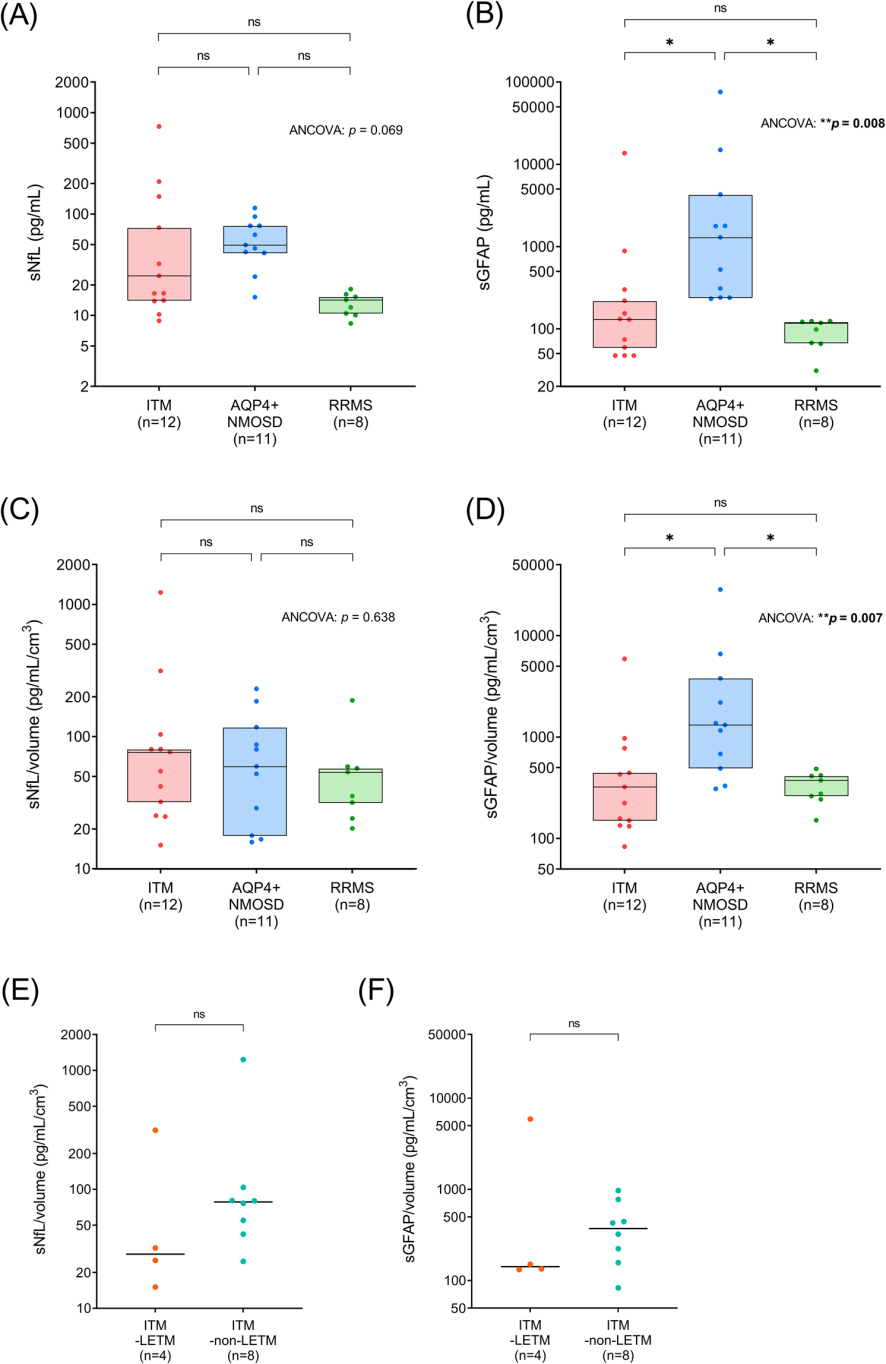
Comparison of serum biomarkers between ITM and other CNS inflammatory demyelinating diseases during acute transverse myelitis attacks. All patients experienced an acute transverse myelitis attack within the last two months before blood sampling. (**A**,**B**) sNfL and sGFAP levels were compared between patients with ITM (n = 12), those with AQP4 + NMOSD (n = 11) and those with RRMS (n = 8). (**C**,**D**) T2 lesion volume-divided sNfL and sGFAP levels were compared between patients with ITM and those with other diseases. The boxes represent medians and interquartile ranges (IQR). (**E**,**F**) T2 lesion volume-divided sNfL and sGFAP were compared between ITM patients with LETM and those without LETM. Bars represent medians. Age was regarded as a covariate in the analyses. *p* values with statistical significance (*p < 0.05 and **p < 0.01) are presented in bold. sNfL: serum neurofilament; sGFAP: serum glial fibrillary acidic protein; ITM: idiopathic transverse myelitis; AQP4 + NMOSD: aquaporin-4 antibody-positive neuromyelitis optica spectrum disorder; RRMS: relapsing–remitting multiple sclerosis; LETM: longitudinally extended transverse myelitis; ANCOVA: analysis of covariance.

sNfL levels during attacks did not differ significantly across the three disease groups (Fig. 4A, p = 0.069), while sGFAP levels did (Fig. 4B, p = 0.008). ITM patients had lower sGFAP levels (130.76 [50.47–280.17] pg/mL) than AQP4 + NMOSD patients (1300.39 [240.16–4293.30] pg/mL, *p* = 0.026), while the sGFAP levels of RRMS patients (108.36 [66.21–123.06] pg/mL) were comparable with those with ITM (*p* > 0.999). In agreement with the observed sNfL and sGFAP patterns, sNfL/volume was comparable across the groups (Fig. 4C, p = 0.638), while sGFAP/volume differed significantly (Fig. 4D, p = 0.007). sGFAP/volume was also lower in ITM patients (273.35 [138.50 – 692.57] pg/mL/cm^3^) compared to AQP4 + NMOSD patients (1317.56 [492.51–3780.30] pg/mL/cm^3^, *p* = 0.011) but comparable between ITM and RRMS patients (325.74 [248.54–417.31] pg/mL/cm^3^, *p* > 0.999). Finally, we compared sNfL/volume and sGFAP/volume between ITM patients with LETM and those with non-LETM and found no significant differences (sNfL/volume: Fig. 4E, p = 0.457, sGFAP/volume: Fig. 4F, p = 0.679).

**Table 3:** Demographic and clinical features of patients with acute transverse myelitis attacks.

	ITM (n = 12)	AQP4 + NMOSD (n = 11)	RRMS (n = 8)	*p* value			
				Multiple comparison	ITM vs. NMOSD	ITM vs. RRMS	NMOSD vs. RRMS
Age, year	45.0 [38.0–50.0]	56.0 [43.0–59.0]	36.0 [23.0–52.0]	**0.016**	0.120	> 0.999	**0.019**
Male	5 (41.7%)	1 (9.1%)	2 (25.0%)	–	0.155	0.642	0.546
Interval since the last attack, days	30.0 [14.0–42.0]	8.0 [5.0–10.0]	10.0 [8.0–45.0]	**0.021**	**0.018**	> 0.999	0.308
EDSS score	3.0 [2.0–4.0]	3.5 [3.0–6.5]	2.5 [2.0–4.0]	0.128	–	–	–
Annual relapse rate/year	–	0.79 [0.46–1.13]	1.17 [0.68–2.75]	–	–	–	0.177
Multiple clinical attacks	0 (0.0%)	9 (81.8%)	8 (100.0%)	–	** < 0.001**	** < 0.001**	0.485
LETM attacks	4 (33.3%)	5 (45.5%)	0 (0.0%)	–	0.680	0.117	**0.045**
Number of segments involved	1.0 [1.0–5.0]	4.0 [2.0–6.0]	2.0 [1.0–2.0]	0.122	–	–	–
T2 lesion volume, mL	0.352 [0.171–2.114]	0.949 [0.487–2.595]	0.308 [0.241–0.445]	0.102	–	–	–
Treatment at sampling	6 (50.0%)	11 (100.0%)	7 (87.5%)	**-**	**0.014**	0.158	0.421

All patients experienced an acute transverse myelitis attack within the last two months before blood sampling. Values represent medians with interquartile ranges or counts with percentages. Treatment includes disease-modifying drugs, corticosteroids, and immunosuppressants. *p* values with statistical significance (*p* < 0.05) are presented in bold.

ITM: idiopathic transverse myelitis; AQP4 + NMOSD: aquaporin-4 antibody-positive neuromyelitis optica spectrum disorder; RRMS: relapsing–remitting multiple sclerosis; EDSS: Expanded Disability Status Scale; LETM: longitudinally extended transverse myelitis.

### Comparison of serum biomarker levels between different CNS inflammatory demyelinating diseases in the remission phase

**Figure 5:**
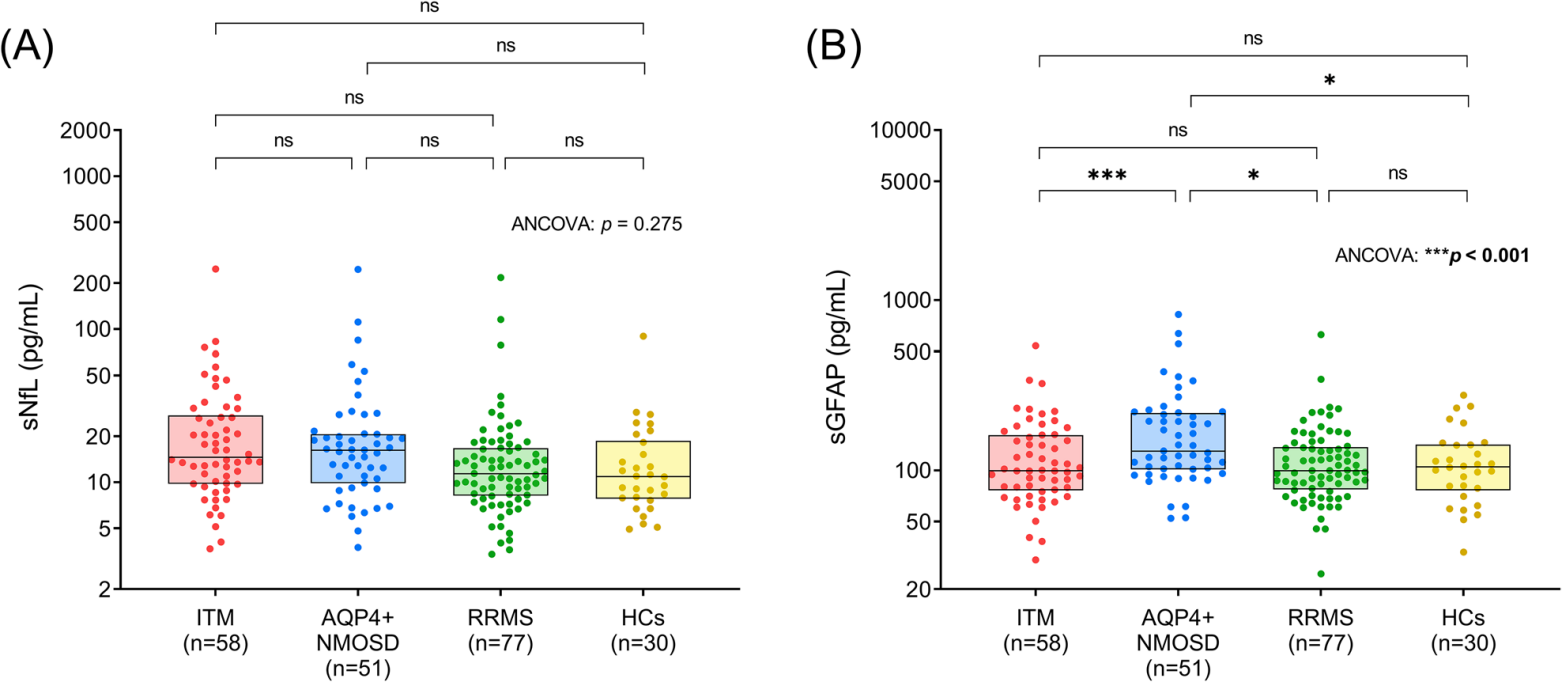
Comparisons of serum biomarkers between patients with ITM, AQP4 + NMOSD and RRMS in remission and HCs. All patients did not experience an acute transverse myelitis attack within the last two months before blood sampling. (**A**) sNfL and (**B**) sGFAP levels were compared between patients with ITM (n = 58), those with AQP4 + NMOSD (n = 51), those with RRMS (n = 77) and HCs (n = 30). The boxes represent medians and interquartile ranges (IQR). Age was regarded as a covariate in the analyses. *p* values with statistical significance (*p < 0.05, **p < 0.01 and ***p < 0.001) are presented in bold. sNfL: serum neurofilament; sGFAP: serum glial fibrillary acidic protein; ITM: idiopathic transverse myelitis; AQP4 + NMOSD: aquaporin-4 antibody-positive neuromyelitis optica spectrum disorder; RRMS: relapsing–remitting multiple sclerosis; LETM: longitudinally extended transverse myelitis; ANCOVA: analysis of covariance.

We also compared serum biomarker levels of patients with ITM, AQP4 + NMOSD and RRMS and HCs during remission phases. sNfL levels did not differ significantly between four groups (Fig. 5A, ANCOVA: *p* = 0.275). sGFAP levels in patients with AQP4 + NMOSD (Fig. 5B, 179.39 [104.75–279.62] pg/mL) were significantly higher than those with ITM (101.10 [73.17–164.08] pg/mL, *p* < 0.001), those with RRMS (99.77 [75.79–134.13] pg/mL, *p* = 0.014) and HCs (104.94 [76.48–142.27] pg/mL, *p* = 0.026).

## Discussion

In this study, we examined sNfL and sGFAP levels in patients with ITM from two perspectives: i) whether sNfL and sGFAP could reflect disease status in ITM as in other CIDDs, and ii) whether there are distinct pathogenetic features of ITM compared to other CIDDs. Both sNfL and sGFAP successfully reflected disease courses, acute attacks, and disease severity. Subsequent investigation with these biomarkers demonstrated that ITM patients experience neuronal and astroglial damage as much as RRMS patients during acute attack, while less astroglial damage than AQP4 + NMOSD patients during both acute attack and remission. Remarkably, compared to HCs, no significant neuronal and astroglial damage was shown in ITM patients during remission, which suggests there is no significant progressive disease characteristic of ITM, at least in this cohort.

Although the exact mechanism is elusive, ITM has been reported to have autoimmune characteristics. Patients with ITM showed elevated interleukin-6 (IL-6) levels in their CSF and pathological findings analogous to those in rat models of spinal cord inflammation^19^. In addition, ITM patients have been found to clinically benefit from plasma exchange and cyclophosphamide treatment^20^. Despite these autoimmune properties, it has often been questioned whether ITM is a separate entity or simply represents incomplete manifestations of known CIDDs^21^. Some clinical data indicate distinct characteristics of ITM, such as male predominance, older onset age, the absence of oligoclonal bands^22,23^, and higher CSF protein levels^11^. Still, there is little direct pathological or serological evidence highlighting differences between ITM and established CIDDs. Moreover, it is even unknown whether neuronal and astroglial damage actually occurs during acute attacks like other CIDDs, and to what extent the damage is severe, if any.

We found that sNfL and sGFAP are significantly increased during acute attacks, and they both were identified as independent factors associated with the acute attack phase of ITM. Meanwhile, in terms of associations between biomarkers and EDSS scores, a statistical significance was shown only between sNfL and EDSS scores, not between sGFAP and EDSS scores. These findings imply that sNfL may be a more sensitive biomarker to assess disability in ITM patients. The property of sNfL and sGFAP to reflect disease activity in ITM is clinically significant because a number of patients with ITM may experience additional attacks. Considering the recurrent^23^ and MRI-negative^24^ TM attack cases, sNfL and sGFAP could serve as useful biomarkers in patients with ITM.

We evaluated sNfL/volume and sGFAP/volume to compare biomarker levels during acute attacks between the disease groups while controlling for lesion volume. This is remarkable because lesion extent could affect serum biomarker levels in CIDDs^12,15^. The three groups of patients (ITM, AQP4 + NMOSD, and RRMS) did not show significant differences in sNfL/volume during acute attacks. These findings suggest that the degree of neuroaxonal damage per lesion volume is comparable across the groups during acute attacks. In contrast, ITM patients showed significantly lower sGFAP/volume than AQP4 + NMOSD patients. Notably, we found that both sNfL/volume and sGFAP/volume did not significantly differ between ITM patients with and without LETM during acute attacks. These findings suggest that the presence of LETM per se does not indicate different pathogenesis such as astrocytopathy^22,25^. Collectively, the characteristics observed in ITM patients were distinguished from those in AQP4 + NMOSD patients but comparable to those in RRMS during acute attacks. Our results also indicate that the pathogenesis of ITM may be distinct from that of AQP4 + NMOSD, whose main mechanism is astrocytopathy.

Another potentially interesting finding from this study is that patients with ITM did not demonstrate evidence of progressive damage process during remission. Patients with ITM in remission showed comparable sNfL and sGFAP levels to HCs, regardless of their lesion extents and multiple attacks. In addition, a significant number of ITM patients remained recurrence-free for a long time (> 10 years) without treatment. It should also be noted that most (96.6%) ITM patients in remission were not receiving corticosteroid or immunosuppressive treatment. Furthermore, it was evident that patients with AQP4 + NMOSD experience more sustained astroglial damage process in their remission compared to patients with ITM, consistent with recent studies^26–28^. However, it should also be noted that MS patients in our cohort also did not demonstrate significantly higher sNfL levels during remission than HCs, unlike the results from other studies^15,27^; thus, our interpretation should be taken cautiously. This discrepancy in patients with MS may have come from unique characteristics of this cohort: (1) relatively milder disease activity or severity in MS patients^15^, (2) lack of patients with progressive MS^27^, and (3) different ethnicity of patients, which may also affect the MS phenotype^29^. Taken together, these findings suggest that most, if not all, patients with ITM, do not have neuroinflammatory damage as a sustained process, especially for astroglial damage.

Several limitations of this study should be discussed. First, because the follow-up period was relatively short, there is a possibility of future conversion from ITM to other CIDDs. Thus, future MS or NMOSD patients may have been incorrectly classified into our ITM group and analyzed as such. Second, although we only included patients whose last attack was transverse myelitis, most patients with RRMS or AQP4 + NMOSD had experienced optic neuritis or demyelinating brain attacks earlier during their disease course, which may have affected their levels of serum biomarkers. However, these patients had long inter-attack intervals between the latest and previous attacks (median [IQR], 542 [174–970] days), which we think should have minimized this potential confounding effect. Also, we only analyzed two biomarkers, focusing on neuroaxonal and astroglial damage. Additional biomarkers reflecting oligodendrocyte damage such as myelin basic protein (MBP), myelin oligodendrocyte glycoprotein (MOG) and proteolipid protein 1 (PLP1) would provide new insights to understand the nature of ITM. In addition, we do not have longitudinal follow-up data within the identical patient. Future studies targeting chronological changes in serum biomarker levels before and after attack or treatment are warranted. Finally, although we recruited consecutive patients with ITM in a prospective manner, it should be noted that we analyzed only a small number of patients from a single center. Studies with data from a larger number of patients are needed, which may unravel additional implications of serum biomarkers in ITM.

Nevertheless, our data show that both sNfL and sGFAP are reliable monitoring biomarkers in ITM. Our analyses indicate that patients with ITM exhibit similar neuronal and astroglial damage compared to those with RRMS but less astroglial damage compared to those with AQP4 + NMOSD during attacks. In addition, it seems that patients with ITM do not appear to experience long-lasting neuroinflammation after remission. These findings suggest that patients with ITM may not share the pathogenesis of MS or NMOSD, which warrants future studies for confirmation.

## Methods

### Patients

We prospectively and consecutively recruited patients with an acute attack or a history of TM who visited a tertiary referral center (Asan Medical Center, Seoul, South Korea) between July 2018 and April 2020. TM attacks were determined when the following criteria were fulfilled^1^: (1) presence of signs or symptoms of sensory, motor, or autonomic dysfunction attributable to the spinal cord; (2) documentation of T2 high signal intensity on spinal MRI. Patients who had experienced clinical attacks within the last two months were regarded as being in an acute attack phase, while all others were regarded as being in the remission phase. For acute attack phase, we only included patients whose last attack was exclusively TM.

A diagnosis of ITM was made based on the previously suggested criteria^30^. All patients underwent a detailed diagnostic workup including brain MRI, spinal MRI, cell-based assaying for anti-aquaporin-4 antibody (AQP4-Ab) and anti-myelin oligodendrocyte glycoprotein antibody (MOG-Ab)^13^, anti-nuclear antibody (ANA), anti-SS-A/SS-B antibody, anti-neutrophil cytoplasmic (ANCA), screening test for HIV and syphilis and routine laboratory test. Patients with evidence of active infections, active malignancy, a history of systemic autoimmune diseases or signs of other various conditions mimicking ITM, such as mechanical compression, spinal cord infarction or spinal arteriovenous fistula were not diagnosed as ITM.

Among the patients with confirmed etiologies, we included AQP4 + NMOSD and RRMS patients for comparison. The diagnoses of AQP4 + NMOSD and RRMS were based on the 2015 International Panel for NMO diagnostic criteria for NMOSD^31^ and the 2017 revised McDonald criteria^32^, respectively. We did not include patients with other etiologies due to small sample sizes. Additionally, healthy controls (HCs), defined as those who complained of mild neurologic symptoms such as headache or dizziness but had normal brain MRI findings, were recruited for further comparison.

All participants were over 18 years of age. Patients were sampled for blood and evaluated for the EDSS score on the day of enrollment.

### Standard protocol approvals, registrations, and patient consent

This study was conducted in accordance with the declaration of Helsinki and approved by the institutional review board of Asan Medical Center (No. 2018-0653). Written informed consent was obtained from all participants.

### Measurements of serum biomarker levels

Serum samples were stored at -80 ℃ according to the standardized protocol^33^ and thawed immediately before the analysis. sNfL and sGFAP were measured in duplicate using a Simoa HD-1 Analyzer (Quanterix, MA, USA) by an investigator blinded to the clinical information. The limit of quantification was 0.241 pg/mL for sNfL and 0.467 pg/mL for sGFAP, compensated for a fourfold sample dilution. All results were above the limit of quantification. The mean intra-assay coefficients of variation for sNfL and sGFAP were 4.9% and 3.1%, respectively, with all coefficients below 20%.

### Lesion volume analysis

Patients with acute TM attacks were evaluated for their myelitis lesion volume in the spinal cord using T2-weighted MRI. To calculate the lesion volume, the hyperintense area of each slice was multiplied by slice thickness using an institutional Digital Imaging and Communications in Medicine (DICOM) viewer (Petavision, Asan Medical Center, Seoul, South Korea)^34^. Longitudinally extensive transverse myelitis (LETM) was defined as myelitis extending over three or more vertebral segments on spinal cord MRI^1^.

### Statistical analysis

Categorical variables were expressed as counts and percentages, and continuous variables as medians and interquartile ranges (IQR). For all analyses, sNfL, sGFAP, sNfL/volume and sGFAP/volume were log-transformed to approximate a normal distribution, whereas results and figures were presented according to their original scales for clarity. Demographic and clinical variables were compared using the Mann–Whitney *U* test, Kruskal-Willis test followed by Dunn-Bonferroni post-hoc pairwise comparison and Fisher’s exact tests. Serum biomarker levels (sNfL, sGFAP, sNfL/volume and sGFAP/volume) were compared using analysis of covariance (ANCOVA) considering age as a covariate, followed by post-hoc main effect comparisons using the Bonferroni method. In all groups, the assumption of equal variance was confirmed using Levene’s test, and the assumption of normality was confirmed using the Kolmogorov–Smirnov or the Shapiro–Wilk test. Although one of the two assumptions was unmet in some groups, all analyses were performed considering the robustness of ANCOVA to single violations^35^ and the exploratory nature of this study.

Associations between serum biomarker levels and clinical variables were tested with linear regression models. For all linear regression analyses, regression coefficients were back-transformed to the original scale (β) and therefore reflected multiplicative effects (i.e., β = 1.01 means an increase of approximately 1% in sNfL or sGFAP). The significance level was set at p < 0.05. All statistical analyses were performed using IBM SPSS version 28.0 (IBM, Armonk, NY, USA). Figures were generated with GraphPad Prism version 9.2.0 (GraphPad Software, San Diego, CA, USA).

## Supplementary Information

Supplementary Figure S1.

## Data Availability

Anonymized data will be available from corresponding author on reasonable request for any qualified investigator.
